# Leveraging Opportunities to Advance the Potential of Technology to Support Independence and Aging in Place

**DOI:** 10.1093/geroni/igaa057.2216

**Published:** 2020-12-16

**Authors:** Tracy Mitzner

**Affiliations:** Georgia Institute of Technology, Atlanta, Georgia, United States

## Abstract

Technology holds great potential to support those aging with and into disability. Research and development efforts in the aging space (aging into disability) have traditionally focused on improving health conditions, whereas those in the disability space (aging with disability) have primarily focused on supporting activity and participation. Bridging these perspectives and approaches adds rich context to guide the development and evaluation of technology interventions. Examples of technology interventions that support activity and participation and thereby improve health outcomes for adults aging with mobility disabilities show the need for bridging. The Telewellness research study used videoconferencing to deliver an evidence-based tai chi intervention to small groups. The Digital Assistant study explored the potential of the Amazon Echo to support controlling the home environment, engaging in physical activity, interacting with others, and managing health. Both projects offer credence to the value of supporting adults aging-in-place with wide range of capabilities and limitations. Part of a symposium sponsored by the Lifelong Disabilities Interest Group.

